# An Anti-Turbulence Self-Alignment Method for SINS under Unknown Latitude Conditions

**DOI:** 10.3390/s22134686

**Published:** 2022-06-21

**Authors:** Yubing Jiao, Jie Li, Xihong Ma, Kaiqiang Feng, Xiaoting Guo, Xiaokai Wei, Yujun Feng, Chenming Zhang, Jingqi Wang

**Affiliations:** National Key Laboratory for Electronic Measurement Technology, North University of China, Taiyuan 030051, China; s2006029@st.nuc.edu.cn (Y.J.); maxihong@nuc.edu.cn (X.M.); b1506011@st.nuc.edu.cn (K.F.); guoxiaoting@nuc.edu.cn (X.G.); b1806023@st.nuc.edu.cn (X.W.); b20210615@st.nuc.edu.cn (Y.F.); sf20210601@st.nuc.edu.cn (C.Z.); s202106010@st.nuc.edu.cn (J.W.)

**Keywords:** SINS, self-alignment, latitude unknown, perturbed environment

## Abstract

For the alignment problem of strapdown inertial navigation system (SINS) under the complex environment of unknown latitude, angular oscillation interference, and line interference, the ant colony simulated annealing algorithm of gravity vector optimization is proposed to obtain the gravity apparent motion vector optimization equation, and the polynomial fitting method is proposed to simultaneously perform latitude estimation and self-alignment in combination with the alignment principle of SINS. Simulations and experiments show that the proposed method has more robust anti-interference capability than the traditional interference-based alignment method, the latitude estimation accuracy is improved by six times, the self-alignment yaw angle error RMSE value after obtaining the latitude is within 0.7°, and the roll angle and pitch angle error values are within 0.1°.

## 1. Introduction

Strapdown inertial navigation system (SINS) is widely used in the fields of aerospace, underwater, and subsurface exploration, with the advantages of full autonomy, strong anti-interference, etc. Alignment is the process of obtaining the initial working state of SINS, which is a necessary prerequisite to ensure the work of SINS, and alignment error is one of the primary error sources of the inertial guidance system, so the alignment accuracy and speed will directly determine the navigation performance and response speed of the system in subsequent work [1]. In general, the initial alignment process requires precise geographic latitude information, which can be obtained directly from satellites; however, in many practical applications, the satellite signal is volatile or even completely absent [2,3]. For fixed bases, the latitude can be estimated from the accelerometer output and gyroscope output, but in the case of interference bases, the inertial device output has a meager signal-to-noise ratio, and when the SINS is applied to geographically uninformative and interference-prone environments, such as mines, tunnels, and deep-sea environments, alignment cannot be achieved directly [4,5]. Therefore, the self-alignment of the jamming base without any external auxiliary equipment is necessary to apply SINS in particular environments.

The classical interference base alignment method based on the gravity apparent motion (GAM) of the inertial system was pioneered by T. Gaiffe et al. [6]. There are two general GAM-based alignment methods. The first is the triad method, which involves construction of three nonlinear vectors using the gravity vectors at two different moments; however, this method is susceptible to random sensor noise and uses only a fraction of the inertial device output [7,8]. The optimization method transforms the determination of the attitude matrix into a Wahba problem and uses the inertial device output in its entirety to achieve higher-accuracy alignment. In 2013, Y. Wu et al. proposed that the optimization-based alignment (OBA) method can achieve interference-based alignment with an optimization algorithm, although the method is not resistant to the effects caused by random interference and environmental noise, and it has limited resistance to interference [9,10].

The initial alignment of SINS in static and perturbed bases under general latitude conditions has been extensively studied in the literature. In the case of uncertain latitude, the local latitude usually has to be calculated first to achieve alignment or to avoid calculating the latitude and thus estimate the carrier attitude directly. With respect to systems in a stationary base, Yan et al. used the angle between the earth rotation angular rate vector and gravity vector to estimate the latitude and used the two-vector attitude fixation method to achieve self-alignment in a stationary base [11]. However, when the system is in perturbed base, the carrier sway rate is much higher than the earth rotation angular rate, and the valid earth rotation angular rate information cannot be extracted from the output of the gyroscope. Another method involves using gradient descent optimization and certain geometric constraints to determine the gravity vector and local latitude in the Earth frame and then constructing the self-alignment process as an optimization-based alignment quaternion determination problem by constructing an objective function using the estimated gravity vector [12]. Wang et al. compared the quantitative, geometric, and analytical methods of latitude determination and introduced a bias estimation method in the latitude determination process. The latitude can be corrected for the navigation latitude of long-term SINS to improve the autonomy and positioning accuracy of the navigation system [13]. Chen et al. projected the gravity vector to the inertial coordinate system, established the relationship between geographic latitude and gravity vector to solve the latitude, and then used the Corsi robust method to construct the objective function to be optimized and introduced the M-estimation theory to realize the self-alignment of the wobble base [14]. There are also methods for introducing fast linear attitude estimators [15]. However, the essence of the above algorithms is to first estimate the latitude value, then, based on the approximate latitude estimate, apply the alignment algorithm under the condition of known latitude to achieve the initial alignment; multiple latitude estimates of the above methods are generally obtained by selecting a few typical points. The statistics of the evaluation results are relatively small, and the output information of the inertial instruments is not fully utilized in real time.

Li et al. bypassed the problem of calculating latitude based on specific geometric constraints of the Earth rate vector in the navigation coordinate system by representing the Earth rate vector with inertial device measurements, although this method is subject to considerable interference from inertial device errors [16,17]. Wang et al. achieved simultaneous latitude self-estimation and coarse alignment using the sliding window method [4], and Zhang et al. artificially reduced the effects of linear vibration interference and measurement noise from inertial sensors [7]. The gravity-based apparent motion observation vector is reconstructed, and Newton’s method is used to optimize and improve the accuracy of the gravity observation vector [18]. Body attitude estimation with unknown latitude is defined as an extended Wahba problem, and latitude determination and body attitude estimation are solved by eigenvalue decomposition based on the inertia tensor of the gravity observation vector; there are also methods of gravity compensation [19], but the principle of this method is more complicated, and the computation is cumbersome, making it difficult to improve the alignment speed [20,21].

Based on the problems in the above algorithms and the fact that the alignment algorithms commonly used in engineering have reached a certain level in terms of running time and accuracy, which are difficult to improve, in this paper, we introduce ant intelligence theory into the alignment problem and proposes a fast self-alignment method based on ant colony intelligence and a polynomial fitting algorithm for real-time latitude estimation of perturbed bases in a latitude-unknown environment. The algorithm is based on an ant colony system and a two-level heuristic algorithm of simulated annealing for real-time optimization of the accelerometer gravity vector, and a polynomial fitting algorithm is used to simultaneously achieve real-time self-alignment of the latitude estimation and attitude matrix calculation. Simulations and experiments show that the algorithm proposed in this paper is effective and can be applied to engineering practice, as it effectively improves alignment speed and alignment accuracy compared with the traditional alignment methods.

The remainder of the paper is organized as follows. In Section 2, the constants, variables, and coordinate systems used in this paper are introduced, followed by the basic principles of alignment, and finally, a general introduction of the proposed method. In Section 3, we mainly describes the principles and steps of the ant colony simulated annealing algorithm and polynomial fitting algorithm proposed in this paper. In Section 4, we simulates and experimentally verify the algorithms proposed in this paper and compare them with other methods.

## 2. Problem Description

The coordinate system used in this paper is described as follows:

*i*-frame: geocentric inertial coordinate system;

*n*-frame: coordinate navigation system; in this paper, the northeast sky geographic coordinate system E-N-U is selected as the navigation reference coordinate system of SINS;

*b*-frame: carrier coordinate system;

$i_{n}$-frame: inertial navigation coordinate system; the coordinate navigation system at the initial moment of alignment is frozen in the inertial system and is stationary with respect to the inertial system during the alignment process;

$i_{b}$-frame: inertial carrier coordinate system; the carrier coordinate system at the initial moment of alignment is frozen in the inertial system and is stationary with respect to the inertial system during the alignment process.

The conversion relations used in this paper are described as follows:

$C_{N}^{M}$: transfer matrix from the *N*-frame to the *M*-frame;

$q_{N}^{M}$: denotes conversion quaternion from the *N*-frame to the *M*-frame.

The constant value matrix used in this paper is described as follows:

(1) $$C_{n}^{i_{n}}=\left[\begin{array}{ccc} \cos\omega_{ie}t & -\sin\omega_{ie}t\sin L & \sin\omega_{ie}t\cos L\\ \sin\omega_{ie}t\sin L & 1-(1-\cos\omega_{ie}t)\sin^{2}L & (1-\cos\omega_{ie}t)\sin L\cos L\\ -\sin\omega_{ie}t\cos L & (1-\cos\omega_{ie}t)\sin L\cos L & 1-(1-\cos\omega_{ie}t)\cos^{2}L \end{array}\right]$$

The differential equation for the specific force of SINS can be described as:

(2) $${\dot{v}}^{n}(t)=C_{b}^{n}(t)f^{b}(t)-\left(2\omega_{ie}^{n}(t)+\omega_{en}^{n}(t)\right)\times v^{n}(t)+g^{n}(t)$$

where $C_{b}^{n}(t)$ is the real-time attitude matrix, $f^{b}$ is the ideal output specific force of the accelerometer, $\omega_{ie}^{n}$ represents the angular rate of rotation of the navigation system due to Earth’s rotation, $\omega_{en}^{n}$ represents the *n*-frame rotation of the inertial guidance system moving near the Earth’s surface due to the curvature of the Earth’s surface and represents the velocity in the *n*-frame, and $g^{n}$ represents the gravity vector in the *n*-frame [22].

When the carrier remains stationary or has only angular motion, the velocity of SINS is 0, which gives:

(3) $$g^{n}=-C_{b}^{n}f^{b}$$

The process of alignment is the process of obtaining the attitude matrix, and at this time, the attitude transformation matrix is chain-decomposed to isolate the interference of angular motion on the attitude matrix, yielding:

(4) $$C_{b}^{n}=C_{i_{n}}^{n}C_{i_{b}}^{i_{n}}C_{b}^{i_{b}}$$

where $C_{i_{n}}^{n}$ is the transformation matrix of the navigation inertial system with respect to the navigation system, $C_{i_{b}}^{i_{n}}$ is the attitude transformation matrix of the carrier inertial system with respect to the navigation inertial system, and $C_{b}^{i_{b}}$ is the transformation matrix of the carrier inertial system with respect to the carrier inertial system. The incorporation of Equation (3) into Equation (4) can be written as:

(5) $$g^{n}=-C_{i_{n}}^{n}C_{i_{b}}^{i_{n}}C_{b}^{i_{b}}f^{b}$$

where $C_{b}^{i_{b}}$ can be updated from the gyroscope real-time output, and $C_{i_{n}}^{n}$ is a constant matrix as above:

(6) $${\dot{C}}_{i_{b}}^{b}=C_{i_{b}}^{b}(\omega_{i_{b}}^{b}\times )$$

**Figure 1:**
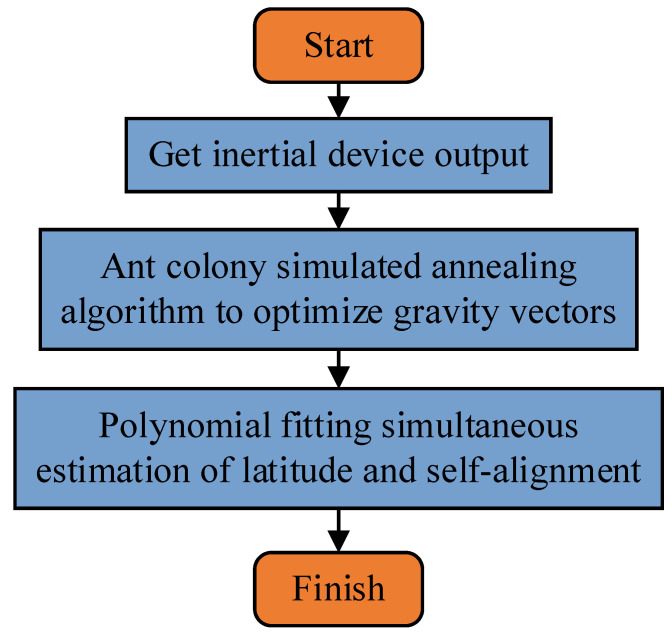
Block diagram of the methodology presented in this paper.

Therefore, Equation (5) can be rewritten by replacing and shifting the terms as follows:

(7) $$f^{i_{b}}=C_{i_{b}}^{i_{n}}F_{g}^{i_{n}}$$

where $F_{g}^{i_{n}}=C_{n}^{i_{n}}g^{n}$, and $f^{i_{b}}$ is the gravitational motion vector described in the $i_{b}$-frame. Optimal estimation of the attitude array can be accomplished by using the equation relationship of all moment vectors of Equation (7) under the condition of known latitude. Then, the attitude array is obtained in real time during the alignment process by combining the calculation results of Equation (5) with the relationship of Equation (6). However, under the condition of unknown latitude, both of the equations cannot be calculated, so we propose an polynomial fitting estimate (PFE) algorithm with time as the independent variable, and latitude estimation and self-alignment are further realized according to the fitting results of the parameters. The specific steps of the method are shown in Figure 1.

## 3. Method

The vector $F_{g}^{i_{n}}$ can be expressed as:

(8) $$F_{g}^{i_{n}}(t)=\left[\begin{array}{c} g\cos L\sin\omega_{ie}t\\ \frac{1}{2}g\sin2L(1-\cos\omega_{ie}t)\\ g\cos^{2}L\cos\omega_{ie}t+g\sin^{2}L \end{array}\right]$$

It is a function with time as the independent variable, for which a power series expansion yields:

(9) $$F_{g}^{i_{n}}(t)=\left[\begin{array}{c} g\cos L\sum_{n=0}^{\infty }\frac{{\left(-1\right)}^{n}}{(2n+1)!}{\left(\omega_{ie}t\right)}^{2n+1}\\ \frac{1}{2}g\sin2L(1-\sum_{n=0}^{\infty }\frac{{\left(-1\right)}^{n}}{(2n)!}{\left(\omega_{ie}t\right)}^{2n})\\ g\cos^{2}L\sum_{n=0}^{\infty }\frac{{\left(-1\right)}^{n}}{(2n)!}{\left(\omega_{ie}t\right)}^{2n}+g\sin^{2}L \end{array}\right],t\in (-\infty ,+\infty )$$

In the polynomial fitting process, the accuracy of latitude and alignment depends mainly on the accuracy of the gravitational apparent motion vector, ${\tilde{f}}^{i_{b}}$. In practice, the actual measurement vector model of the gravitational apparent motion vector, ${\tilde{f}}^{i_{b}}$, can be expressed as:

(10) $${\tilde{f}}^{i_{b}}=C_{b}^{i_{b}}{\tilde{f}}^{b}$$

${\tilde{f}}^{b}$ is the actual measured value of the accelerometer, expressed as:

(11) $${\tilde{f}}^{b}=f^{b}+\nabla_{b}+\eta_{b}$$

where $f^{b}={\left[\begin{array}{ccc} f_{x} & f_{y} & f_{z} \end{array}\right]}^{T}$ is the ideal output ratio of the accelerometer in the b-frame, $\nabla^{b}={\left[\begin{array}{ccc} \nabla_{x} & \nabla_{y} & \nabla_{z} \end{array}\right]}^{T}$ is the deviation vector of the accelerometer, and $\eta^{b}={\left[\begin{array}{ccc} \eta_{x} & \eta_{y} & \eta_{z} \end{array}\right]}^{T}$ is the white noise. According to [18], the equation can be obtained in quadratic form according to Equation (11) as follows:

(12) $${\tilde{f}}^{i_{b}}={\tilde{q}}_{b}^{i_{b}}⊗(f^{b}+\nabla^{b}+\eta^{b})⊗({\tilde{q}}_{b}^{i_{b}})*$$

When the system is at rest or shaking, it can be obtained from Equation (12):

(13) $${\tilde{f}}^{i_{b}}=f^{i_{b}}+\left[\varepsilon^{b}t\times \right]g^{i_{b}}+\nabla^{i_{b}}+\eta^{i_{b}}$$

where $\varepsilon^{b}={\left[{\begin{array}{ccc} \varepsilon_{x} & \varepsilon_{y} & \varepsilon \end{array}}_{z}\right]}^{T}$ describes the bias of the gyroscope. Equation (13) shows that the true value of the gravitational apparent motion vector is not easy to extract, and it is necessary to fit the data output from the inertial device when performing the polynomial fit. The magnitude of the error of the inertial device output determines the accuracy of the self-alignment. In particular, the accelerometer output has an important role in determining the latitude estimation and self-alignment process because it is disturbed by line vibrations, device bias, and environmental factors; therefore, in terms of latitude, the gravitational apparent motion vector needs some processing before estimation and self-alignment.

### 3.1. GAM Vector Optimization Method

The alignment accuracy of the polynomial fit based on the gravitational apparent motion vector relies heavily on the accuracy of the accelerometer output; however, the resulting specific force output is not accurate due to random noise, sensor noise, and interference from line vibration under swing conditions, so the accelerometer output should be optimized prior to alignment. Common optimization methods in inertial device data processing include the Newtonian iterative method and gradient descent method, but these methods are computationally intensive, resulting in long computation time and difficulty of use in practical engineering fields. Therefore, we propose a method involving an ant colony simulated annealing algorithm to optimize the gravitational apparent motion vector.

The ant colony algorithm is essentially a positive feedback mechanism, but the pheromone generated during the initial iteration is very limited, so the solution speed is slow, and it is easy to fall into the problem of local optimum in the later stage. Therefore, a simulated annealing algorithm is introduced on the basis of the ant colony algorithm [23,24,25,26]. The annealing algorithm generates an optimal solution quickly with a certain probability criterion, so it effectively addresses the problem of the ant colony algorithm falling into local optimum, and it can provide additional pheromones for the ant colony so that the whole optimization process converges faster, and the ant colony algorithm can solve the problem of simulated annealing having no feedback mechanism and low solution accuracy [27].

In order to obtain more accurate optimization results, an improved adaptive artificial ant colony (ACC) algorithm—ant colony system (ACS)—is applied here. Compared with the traditional artificial ant colony algorithm, this algorithm combines distributed computing, positive feedback mechanisms, and greedy search, which has the advantages of strong parallelism and better solution searchability and is beneficial for practical applications. Therefore, in this paper, the ant colony system and simulated annealing algorithm are referred to the optimization problem of gravitational apparent motion vector, and the two are combined, compensating for each other to achieve the optimal alignment accuracy and the fastest alignment speed.

The truth-value model of the gravitational apparent motion vector can be written according to Equation (10):

(14) $$f^{i_{b}}=\;C_{b}^{i_{b}}f^{b}=C_{i_{e}}^{i_{b}}C_{e}^{i_{e}}C_{n}^{e}(-g^{n})$$

It is the constant value matrix from the $i_{e}$-frame to the $i_{b}$-frame during the alignment process. When SINS is at rest or in a state with only angular motion, $C_{n}^{e}$ is the constant value matrix from the *n*-frame to the *e*-frame. The transformation equation can be written as:

(15) $$f^{i_{b}}=C_{i_{n}}^{i_{b}}\left[\begin{array}{ccc} 0 & g\cos L & 0\\ -g\sin L\cos L & 0 & g\sin L\cos L\\ g\cos^{2}L & 0 & g\sin^{2}L \end{array}\right]\left[\begin{array}{c} \cos\omega_{ie}t\\ \sin\omega_{ie}t\\ 1 \end{array}\right]$$

The actual gravitational apparent motion vector variation frequency, $\omega_{ie}/2\pi \approx 1.1574\times 10^{-5}\;\mathrm{Hz}$, is a slow-varying vector, and becuase the components of the $C_{i_{n}}^{i_{b}}$ matrix are constant, the true value model of the gravitational apparent motion vector can be written as:

(16) $$f^{i_{b}}=A{\left[\begin{array}{ccc} \cos\omega_{ie}t & \sin\omega_{ie}t & 1 \end{array}\right]}^{T}$$

where *A* is a 3 × 3 matrix used to represent the optimal constructed matrix so that the actual gravitational apparent motion vector is closest to the ideal gravitational apparent motion vector. The equation reflects the inconsistency error between the theoretical and measured values, and the matrix, *A*, can be optimized according to the following equation so that the value of the function, *F*(*A*), at each moment, *t*, is closest to the minimum value:

(17) $$F(A)={\left|f^{i_{b}}(t)-{\tilde{f}}^{i_{b}}(t)\right|}^{2}$$

The ant colony simulated annealing (ACSA) algorithm is based on the ant colony system (ACS), and the simulated annealing algorithm mechanism is introduced to optimize the actual gravitational apparent motion vector at each moment, *t*. Assume that the number of ants is *m*; the ant colony search is restricted to the *edge*; the number of iterations for the colony to find the optimal value is *n*; the initialized ant colony, $a_{0}$, is randomly distributed in the feasible domain; $\tau_{i}$ indicates the pheromone concentration on the random solution, $F_{i}$, where the ants are located; the pheromone concentration at the initial moment, $\tau_{0}$, is indicated by the function value *D*_0_, which is derived from the initial ant colony; *T*_0_ is the initial temperature of simulated annealing; *T*_min_ is the minimum temperature of the annealing process; and *r* is the cooling factor. The smaller the function value in this algorithm, the higher the ant colony pheromone concentration.

The transfer probability parameter, *p*_0_, is introduced and compared with the state transfer probability to determine the transfer state of the colony, and the state transfer probability can be determined by the following equation:

(18) $$p_{i}=\left|\left(\max(\tau_{0})-\tau_{i})/\max(\tau_{0}\right)\right|$$

An ant colony smaller than the transfer probability parameter only conducts local search according to prior knowledge, and the rest of the ant colony conducts global search, this operation avoids prevents the whole search process from falling into local optimum. The transfer state, $T_{ij}$, can be determined by Equation (19):

(19) $$T_{ij}=\;\{\begin{array}{l} a_{i}+(2*rand-1)/n,p_{i}<p_{0}\\ a_{i}+edge*(rand-0.5),p_{i}\geq p_{0} \end{array}$$

Instead of updating the pheromone for all ants in the ant colony system, only the best ant in each cycle releases the pheromone, which makes the whole process of finding the best faster. Pheromones are updated by:

(20) $$\tau_{j}=(1-\rho )\tau_{i}+\rho \Delta \tau_{i}$$

(21) $$\Delta \tau_{i}=\{\begin{array}{c} 1/F_{i},\min F_{i}\\ 0,\mathrm{otherwise} \end{array}$$

where ρ∈(0,1) is the information volatility factor, and $\Delta \tau_{i}$ is the relevant parameter determined by the optimal ant. With the above method, the optimal convergence of matrix *A* is completed. When the ant colony completes a round of search, random oscillations are added to the current optimal ant as a benchmark to generate a new solution, *D_k_*, which is accepted if the new solution is smaller—otherwise, with a certain probability. The acceptance probability is:

(22) $$q_{k}=\;\{\begin{array}{l} \exp(-\Delta D/T_{k}),\mathrm{otherwise}\\ 1,\Delta D\leq 0 \end{array}$$

where Δ*D* = *D_k_* − *F_k_* denotes the difference between the new solution and the optimal ant, and *T_k_* is the current temperature. When the simulated annealing inner cycle and the cooling operation are completed, the cooling equation is:

(23) $$T_{k}=T_{k-1}\cdot r$$

Let $\hat{A}$ be the optimal solution. The gravity vector model can be rewritten as:

(24) $${\hat{f}}^{i_{b}}=\hat{A}{\left[\begin{array}{ccc} \cos\omega_{ie}t & \sin\omega_{ie}t & 1 \end{array}\right]}^{T}$$

**Figure 2:**
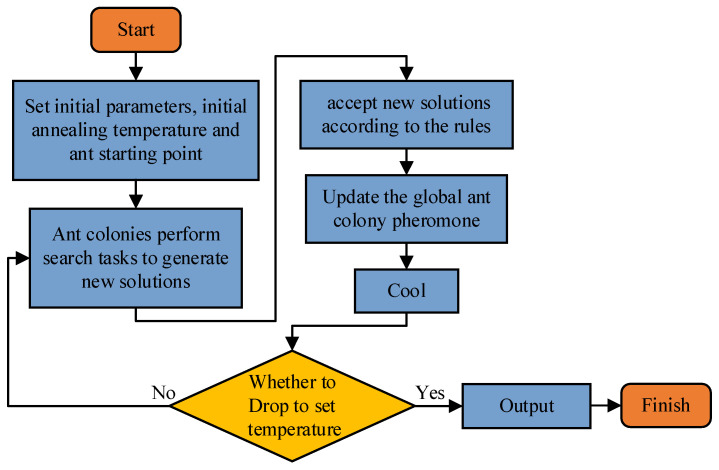
Flow chart of artificial ant colony simulated annealing algorithm.

In (7) $f^{i_{b}}$ is replaced by the more accurate gravity vector, ${\hat{f}}^{i_{b}}$, for subsequent calculations, which improves the alignment accuracy to a certain extent. Figure 2 shows a flow chart of the ant colony simulated annealing two-layer heuristic algorithm.

### 3.2. Alignment Method for Polynomial Fitting

The fitting process has a quadratic smoothing effect on the integration error of the disturbance acceleration, and further latitude estimation and self-alignment can be achieved based on the fitting results of the parameters.

$C_{i_{n}}^{i_{b}}$ can be expressed in matrix form as:

(25) $$C_{i_{n}}^{i_{b}}=\left[\begin{array}{ccc} k_{11} & k_{12} & k_{13}\\ k_{21} & k_{22} & k_{23}\\ k_{31} & k_{32} & k_{33} \end{array}\right]$$

Expanding Equation (9) gives:

(26) $$f^{i_{b}}(t)=\left[\begin{array}{c} \begin{array}{l} k_{11}g\cos L(\omega_{ie}t-\frac{1}{6}{\left(\omega_{ie}t\right)}^{3}+\cdots +\frac{{\left(-1\right)}^{n}}{(2n+1)!}{\left(\omega_{ie}t\right)}^{2n+1})+\frac{1}{2}k_{12}g\sin2L(\frac{1}{2}{\left(\omega_{ie}t\right)}^{2}+\cdots +\frac{{\left(-1\right)}^{n}}{(2n)!}{\left(\omega_{ie}t\right)}^{2n})\\ +K_{13}g\cos^{2}L(1-\frac{1}{2}{\left(\omega_{ie}t\right)}^{2}+\cdots +\frac{{\left(-1\right)}^{n}}{(2n)!}{\left(\omega_{ie}t\right)}^{2n})+k_{13}g\sin^{2}L \end{array}\\ \begin{array}{l} k_{21}g\cos L(\omega_{ie}t-\frac{1}{6}{\left(\omega_{ie}t\right)}^{3}+\cdots +\frac{{\left(-1\right)}^{n}}{(2n+1)!}{\left(\omega_{ie}t\right)}^{2n+1})+\frac{1}{2}k_{22}g\sin2L(\frac{1}{2}{\left(\omega_{ie}t\right)}^{2}+\cdots +\frac{{\left(-1\right)}^{n}}{(2n)!}{\left(\omega_{ie}t\right)}^{2n})\\ +K_{23}g\cos^{2}L(1-\frac{1}{2}{\left(\omega_{ie}t\right)}^{2}+\cdots +\frac{{\left(-1\right)}^{n}}{(2n)!}{\left(\omega_{ie}t\right)}^{2n})+k_{23}g\sin^{2}L \end{array}\\ \begin{array}{l} k_{31}g\cos L(\omega_{ie}t-\frac{1}{6}{\left(\omega_{ie}t\right)}^{3}+\cdots +\frac{{\left(-1\right)}^{n}}{(2n+1)!}{\left(\omega_{ie}t\right)}^{2n+1})+\frac{1}{2}k_{32}g\sin2L(\frac{1}{2}{\left(\omega_{ie}t\right)}^{2}+\cdots +\frac{{\left(-1\right)}^{n}}{(2n)!}{\left(\omega_{ie}t\right)}^{2n})\\ +K_{33}g\cos^{2}L(1-\frac{1}{2}{\left(\omega_{ie}t\right)}^{2}+\cdots +\frac{{\left(-1\right)}^{n}}{(2n)!}{\left(\omega_{ie}t\right)}^{2n})+k_{33}g\sin^{2}L \end{array} \end{array}\right]$$

By collation, we can obtain:

(27) $$f^{i_{b}}(t)\;=\;\left[\begin{array}{c} (k_{13}g)+(k_{11}g\omega_{ie}\cos L)t+(\frac{1}{4}k_{12}g\omega_{ie}{}^{2}\sin2L-\frac{1}{2}k_{13}g\omega_{ie}{}^{2}\cos^{2}L)t^{2}+(-\frac{1}{6}k_{11}g\omega_{ie}{}^{3}\cos L)t^{3}+\cdots \\ (k_{23}g)+(k_{21}g\omega_{ie}\cos L)t+(\frac{1}{4}k_{22}g\omega_{ie}{}^{2}\sin2L-\frac{1}{2}k_{23}g\omega_{ie}{}^{2}\cos^{2}L)t^{2}+(-\frac{1}{6}k_{21}g\omega_{ie}{}^{3}\cos L)t^{3}+\cdots \\ (k_{33}g)+(k_{31}g\omega_{ie}\cos L)t+(\frac{1}{4}k_{32}g\omega_{ie}{}^{2}\sin2L-\frac{1}{2}k_{33}g\omega_{ie}{}^{2}\cos^{2}L)t^{2}+(-\frac{1}{6}k_{31}g\omega_{ie}{}^{3}\cos L)t^{3}+\cdots \end{array}\right]$$

Modeling the above equation in polynomial form for time *t* yields:

(28) $$\left\{ \begin{array}{l} f_{x}^{i_{b}}(t)=a_{x0}t^{0}+a_{x1}t^{1}+a_{x2}t^{2}+a_{x3}t^{3}+\cdots +a_{xn}t^{n}\\ f_{y}^{i_{b}}(t)=a_{y0}t^{0}+a_{y1}t^{1}+a_{y2}t^{2}+a_{y3}t^{3}+\cdots +a_{yn}t^{n}\\ f_{z}^{i_{b}}(t)=a_{z0}t^{0}+a_{z1}t^{1}+a_{z2}t^{2}+a_{z3}t^{3}+\cdots +a_{zn}t^{n} \end{array}\right.$$

Comparing Equations (27) and (28) yields:

(29) $$\left[\begin{array}{c} a_{x0}\\ a_{y0}\\ a_{z0} \end{array}\right]=\left[\begin{array}{c} k_{13}g\\ k_{23}g\\ k_{33}g \end{array}\right]$$

(30) $$\left[\begin{array}{c} a_{x1}\\ a_{y1}\\ a_{z1} \end{array}\right]=\left[\begin{array}{c} k_{11}g\omega_{ie}\cos L\\ k_{21}g\omega_{ie}\cos L\\ k_{31}g\omega_{ie}\cos L \end{array}\right]$$

Modulo the two ends of Equations (29) and (30), and combining the orthogonal properties of the attitude array results in the following latitude:

(31) $$\left|\hat{L}\right|=\arccos\frac{\sqrt{a_{x1}{}^{2}+a_{y1}{}^{2}+a_{z1}{}^{2}}}{\omega_{ie}\sqrt{a_{x0}{}^{2}+a_{y0}{}^{2}+a_{z0}{}^{2}}}$$

According to Equations (29) and (30), combining the orthogonal properties of the attitude array affords:

(32) $$\left[\begin{array}{c} k_{12}\\ k_{22}\\ k_{32} \end{array}\right]=\frac{1}{\left\|a_{1}\right\|\cdot \left\|a_{0}\right\|}\left[\begin{array}{c} a_{x1}\\ a_{y1}\\ a_{z1} \end{array}\right]\times \left[\begin{array}{c} a_{x0}\\ a_{y0}\\ a_{z0} \end{array}\right]$$

The solution of the attitude array, $C_{b}^{n}$, is completed by Equations (1) and (25). According to the feature that the sign of the northward projection component of gravity under the initial navigation inertial system is the same as the sign of latitude, the positive and negative signs of latitude are solved quickly, as well as the estimated value of latitude.

## 4. Simulation and Experiment

### 4.1. Simulation Test

Simulation experiments were conducted to verify the feasibility of the wobble base self-alignment algorithm under the unknown latitude conditions proposed in this paper. Algorithm data are processed using the MATLAB platform under the CPU computer system, and the simulation experiments are set as follows: the geographic location information is 38.02° latitude and 112.46° longitude. The simulation process assumes that the inertial devices of the three axes are identical, and their parameters are set as shown in Table 1. The wobble signal is added to the inertial navigation system, Asin(ωt+φ)+B. The wobble parameters of each axis are set as shown in Table 2. The reference true value in the simulation experiment is taken as the standard. The system alignment time is 300 s, and the sampling rate is 100 Hz.

#### 4.1.1. Gravity Vector Optimization

**Figure 3:**
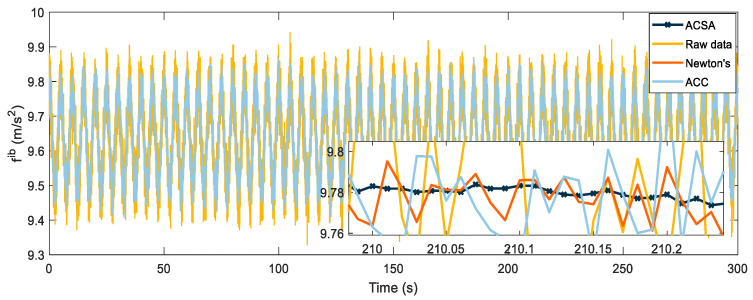
Gravity vector optimization result graph.

In order to verify the effectiveness of the method proposed in this paper and simulate its application environment more realistically, white noise with a mean value of 0 and variance of 0.002 is added to the output signal of the inertial device in the following simulations. The gravity vector data are compared with the original data after being processed by an artificial ant colony. Newton’s method and the ant colony presented in this paper simulated an annealing two-layer algorithm. Results are presented in Figure 3.

The original data with noise added fluctuate considerably, and the gravity vector at each moment differs from its true value. The effect of noise removal can be achieved by simulated annealing and the artificial ant colony algorithm, but the denoising result of the optimized data is not satisfactory, and the data following the ant colony simulated annealing double layer algorithm is smoother and closer to the true value. In order to compare the advantages and disadvantages of the three methods more intuitively, each calculates its root mean square error (RMSE) value, which is used to measure the deviation between the observed value and the true value. The RMSE is defined as:

(33) $$re=\sqrt{\frac{\sum d_{i}}{n}}$$

where *re* represents the value of RMSE, *d_i_* represents the deviation of a set of measurements from the true value, and *n* is the number of measurements. The RMSE values of the three algorithms and the original data are shown in Table 3; the lower their values, the fewer the errors and the closer to the true values.

The RMSE value of ACSA is only 0.0014, which is an order of magnitude lower than the RMSE value of the gravity vector solved by the original data. Its accuracy is improved by a factor of five compared to the Newton iterative method and nearly ten times better than the artificial ant colony algorithm. We also compared the running time of the above algorithms, and Table 4 presents the optimization time of each algorithm during the 300 s alignment process.

Newton’s has the longest run time, and ACSA has the shortest run time, reducing the run time by one-fifth.

#### 4.1.2. Latitude Estimation

**Figure 4:**
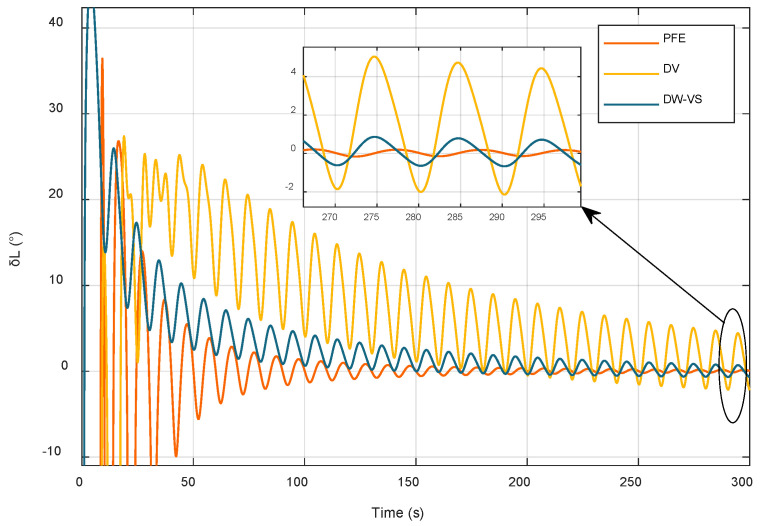
Comparison of latitude estimation methods.

In order to verify the effectiveness of the method and PFE presented in this paper, the traditional double-vector (DV) clip angle fixed latitude method and the sliding window self-estimation algorithm (DW-VS) are compared by simulation experiments, and the comparison results are shown in Figure 4.

All three methods proposed above can achieve the self-estimation of latitude; however, due to the influence of base wobble, the traditional two-vector fixed latitude method converges slowly and with low accuracy. Although the sliding window method converges faster in the initial stage of alignment, the convergence speed of the alignment result increases with the increase in the alignment time, and the convergence speed of the alignment result is not obvious. Compared with the other two algorithms, the convergence speed of the algorithm proposed in this paper increases significantly with increased alignment time, and the accuracy of the final latitude estimation results is higher. In order to further compare the advantages and disadvantages of the three methods, the first-order fitting algorithm is used as an example, and the mean and root mean square error of their respective latitude errors at different time periods are calculated to compare their algorithm performance. Comparison results are presented in Table 5.

Table 5 shows that in the time interval of 0–100 s, the RMSE values of the three methods do not differ considerably; in the time interval of 101–200 s, the value of RMSE of the dual vector method is 16 times higher than that of the algorithm proposed in this paper, and the sliding window method is better in comparison. However, the RMSE value is three times higher than that of the algorithm proposed in this paper. In the 201–300 s interval, the algorithm presented in this paper achieves the best performance, which is 13 times and 3 times better than that of the dual vector method and the sliding window method, respectively. In the interval of 201–300 s, the proposed algorithm outperforms the two-vector method and the sliding window method by a factor of 13 and 3, respectively.

**Figure 5:**
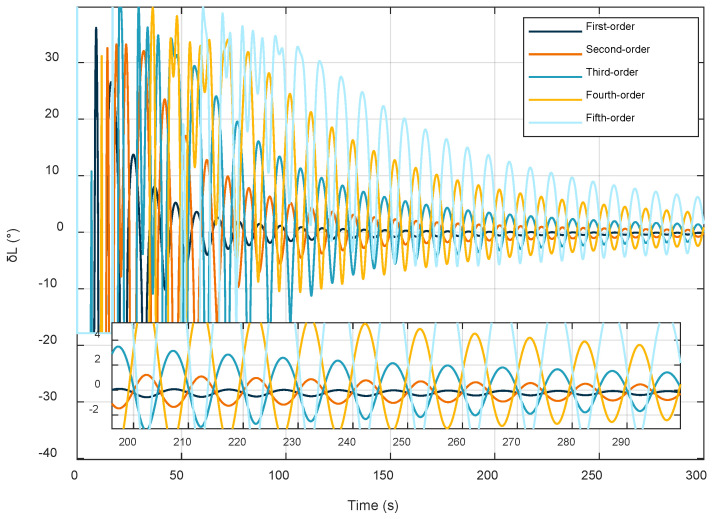
Fitting results of different orders.

The effect of different fitting orders on the latitude estimates. The latitude estimation errors at different orders are shown in Figure 5.

As shown above, the degree of fit gradually improves with increasing time, but the larger the order of fit, the worse the convergence of the latitude estimation results. The fit results cannot be evaluated by this criterion alone, so Table 6 shows the fit errors for different time intervals.

According to the results in the table, in the time interval of 0–100 s, the errors of the first-order fit, the second-order fit, and the fifth-order fit do not differ considerably and perform better; in the time interval of 101–200 s, the results of the third-order fit are improved compared with the previous time period; the results of the first-order fit and the second-order fit are improved, but the effect is not obvious; and the results of the fourth-order fit and the fifth-order fit are worse than the previous time period. In the time interval of 201–300 s, the results of third-order fitting, fourth-order fitting, and fifth-order fitting are improved compared with the previous time period, and the results of first-order fitting and second-order fitting are worse than in the previous time period. In summary, the low-order fit performs well in the initial stage of the fit, but the fitting effect increases with the fitting time. Unlike the other orders at the same time, the high-order fit fluctuates more in the initial stage of the fit, but the fitting effect gradually improves with increased time, and considering the above factors, the third-order fit performs best, so the third-order fit is used for latitude estimation in this paper.

#### 4.1.3. Alignment

**Figure 6:**
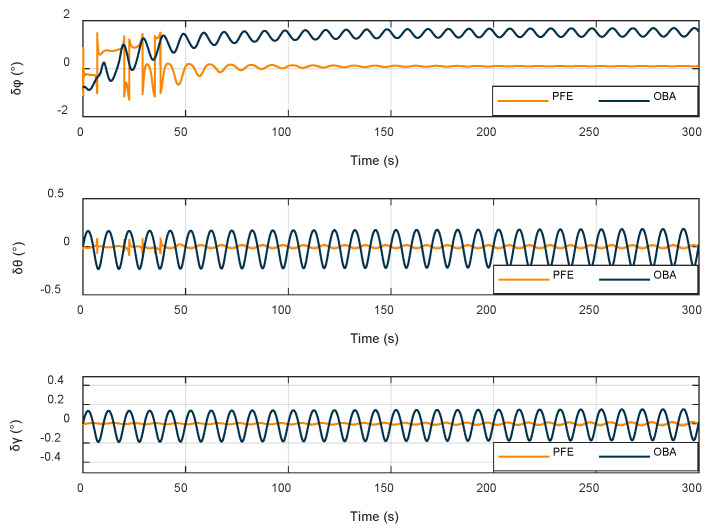
Attitude angle alignment errors.

In order to verify the effectiveness of the alignment method proposed in this paper, the method PFE is compared with the OBA method, and the related results are analyzed. Because the OBA method cannot be implemented under the unknown latitude condition, this section is related to the latitude estimation results presented in the previous section. Figure 6 shows the alignment error of attitude angle, where *φ* denotes the yaw angle, *γ* denotes the roll angle, and *θ* denotes the pitch angle.

As shown in the Figure 6, both methods can achieve the effect of alignment, but the method proposed in this paper obviously has a better convergence effect compared with the OBA method and achieves higher accuracy with respect to the alignment of yaw angle. In order to further compare the effectiveness of the two methods, their RMSE values are calculated for comparison in Table 7.

The alignment method proposed in this paper is more effective than OBA, in which the alignment accuracy of all three attitude angles is improved by an order of magnitude.

### 4.2. Experimental Validation

**Figure 7:**
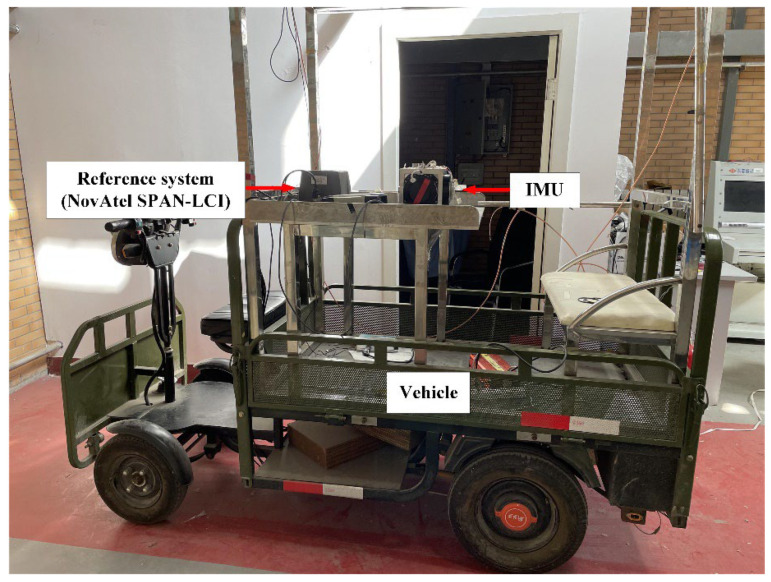
Vehicle experimental setup.

In order to verify the effectiveness of the method proposed in this paper, a set of in-vehicle experiments are designed to simulate the interference environment of SINS by artificially shaking the vehicle. Figure 7 shows the vehicle experiments, and Table 8 lists the parameters of the IMU used in the experiment. Table 9 lists the parameters of the reference system.

**Figure 8:**
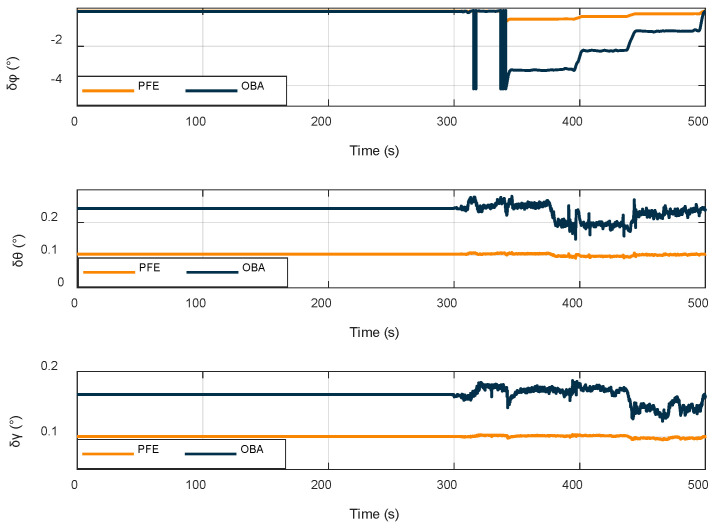
Alignment attitude angle error.

The whole alignment process takes 500 s. The first 300 s are stationary time, and then the vehicle is shaken randomly every 50 s. The performance of roll angle, pitch angle, and yaw angle in the IMU experiment is evaluated using the reference system as the true value. Figure 8 shows the attitude angle error of the experimental alignment.

Figure 8 shows that the anti-interference effects of the OBA and PFE methods do not differ considerably when they are stationary, and the surrounding environment is only mildly disturbed; when large and complex interference is artificially added, the alignment curve of the OBA method starts to show random dispersion, whereas the PFE method is less affected by the interference. In order to further compare the two methods, the RMSE values, as well as the maximum and minimum values of each moment, are listed in Table 10 for further comparison.

According to analysis of the RMSE data, the alignment performance of the OBA and PFE models does not differ when the SINS is stationary; however, after the vehicle is in motion, the OBA alignment accuracy decreases significantly, and the algorithm proposed in this paper produces only a small change, and the alignment accuracy is improved by an order of magnitude compared with the OBA algorithm. Furthermore, the alignment error of yaw angle ranges from 0.2 to 0.7°, and the roll angle and pitch angle alignment errors are within 0.1°.

## 5. Conclusions

In this paper, with respect to the problem of self-alignment of the SINS interference base, a two-layer heuristic optimization algorithm with artificial ant colony simulated annealing is used for gravity vector processing, which effectively improves the ability of global search, avoids the problem of falling into local optimum, makes the subsequent alignment process more accurate, and improves the computing speed of the whole alignment process. The polynomial fitting method is then used to solve the latitude estimation and attitude array simultaneously. Simulation tests and onboard physics experiments show that compared with the traditional perturbed-base method, the method proposed in this paper has a simple principle, high accuracy of latitude estimation, and strong anti-interference capability, making it easy to apply in engineering practice.

## Figures and Tables

**Table 1:** Simulated inertial device parameters.

Gyroscope		Accelerometer	
Constant value (deg/h)	$\mathrm{Random}\;(\deg/\sqrt{h}$)	Constant value (ug)	$\mathrm{Random}\;(\mathrm{ug}/\sqrt{\mathrm{Hz}}$)
0.01	0.001	100	10

**Table 2:** Simulated inertial device sway parameters.

Parameter		X	Y	Z
A (°)		10	10	10
ω (°/s)		π/5	π/5	π/5
φ (°)		0	0	0
B (mm)	t = 2n (*n* = 0,1,…)	1	1	1
	t = 2n + 1 (*n* = 0,1,…)	−1	−1	−1

**Table 3:** Gravity vector RMSE values.

Method	Raw Data	Newton’s	ACC	ACSA
RMSE	0.0449	0.0064	0.0103	0.0014

**Table 4:** Gravity vector optimization time.

Method	Newton’s	ACC	ACSA
Running time (s)	15.86	13.03	4.77

**Table 5:** Comparison of results of latitude estimation methods.

Method	Time Interval/s	Mean/°	RMSE/°
PFE	[0, 100]	−1.2352	10.4168
	[101, 200]	−0.2294	0.5661
	[201, 300]	−0.2327	0.2857
DV	[0, 100]	10.0973	18.2385
	[101, 200]	6.8325	7.9523
	[201, 300]	2.4220	3.6445
DW-VS	[0, 100]	9.2512	13.9538
	[101, 200]	1.4002	1.7734
	[201, 300]	0.2868	0.6718

**Table 6:** Mean values of latitude errors of different fitting orders.

Fitting Order	Time Interval/s	Error Mean/°
1	[0, 100]	−1.2352
	[101, 200]	−0.2294
	[201, 300]	−0.2327
2	[0, 100]	−0.1867
	[101, 200]	0.0933
	[201, 300]	−0.1627
3	[0, 100]	3.0752
	[101, 200]	0.8635
	[201, 300]	0.1130
4	[0, 100]	3.5412
	[101, 200]	4.7225
	[201, 300]	1.0349
5	[0, 100]	−0.9813
	[101, 200]	12.0035
	[201, 300]	3.1203

**Table 7:** Evaluation of alignment error RMSE values.

Method	Posture Angle	RMSE (°)
OBA	Yaw	1.3572
	Roll	0.1394
	Pitch	0.1140
PFE	Yaw	0.1779
	Roll	0.0131
	Pitch	0.0082

**Table 8:** Parameters of IMU.

Gyro Bias	Gyro Random Walk	Accelerometer Bias	Frequency
1°/h	$0.5{}^{\circ}/\sqrt{h}$	10 mg	50 Hz

**Table 9:** Parameters of reference system.

Position Accuracy	Velocity Accuracy	Time Accuracy	Frequency
1 cm ± 1 ppm	0.03 m/s	20 ns	100 Hz

**Table 10:** Performance comparison of alignment methods.

Method	Time/s	Posture	RMSE (°)	Max (°)	Min (°)
OBA	[0, 300]	Yaw	0.2247	−0.2178	−0.2199
		Pitch	0.1726	0.1713	0.1712
		Roll	0.1652	0.1647	0.1645
	[301, 500]	Yaw	2.3672	0.1700	−4.1690
		Pitch	0.2654	0.1901	0.1245
		Roll	0.2637	0.1861	−0.1236
PFE	[0, 300]	Yaw	0.2066	−0.2065	−0.2067
		Pitch	0.1015	0.1052	0.1015
		Roll	0.1003	0.1007	0.1006
	[301, 500]	Yaw	0.4400	−0.2002	−0.6930
		Pitch	0.0986	0.0997	0.0952
		Roll	0.0994	0.0984	0.0948

## Data Availability

Not applicable.
